# Safety incidents associated with extended working hours. A systematic review and meta-analysis

**DOI:** 10.5271/sjweh.3958

**Published:** 2021-08-31

**Authors:** Dagfinn Matre, Marit Skogstad, Tom Sterud, Karl-Christian Nordby, Stein Knardahl, Jan Olav Christensen, Jenny-Anne S Lie

**Affiliations:** National Institute of Occupational Health, Oslo, Norway

**Keywords:** Key terms accident, injury, long working time, long work week, near accident, occupational injury, overtime, safety-related incident

## Abstract

**Objective:**

We performed a systematic review to assess potential consequences of extended working hours on accidents, near-accidents, safety incidents and injuries (hereafter ’incidents’) by considering the overall certainty of evidence.

**Methods:**

We searched five databases systematically (Medline, Embase, PsycINFO, Web of Science, and Proquest Health and Safety Science Abstracts) and identified 10 072 studies published up to December 2020, 22 of which met the inclusion criteria. We followed a systematic approach to evaluate risk of bias and synthesize results in a meta-analysis. The certainty of evidence was determined by a modified version of the Grading of Recommendations Assessment, Development and Evaluation (GRADE).

**Results:**

Our analyses indicated an association between working >12 hours/day [relative risk (RR) 1.24, 95% confidence interval (CI) 1.11–1.40], or working >55 hours/week (RR 1.24, 95% I 0.98–1.57), and elevated risk of incidents. The certainty of evidence evaluated as low. Weak or no associations were observed for other exposure contrasts: working >8 hours/day (RR 0.93, 95% CI 0.72–1.19), or working overtime (RR 1.08, 95% CI 0.75–1.55), working 41–48 hours/week (RR 1.02, 95% CI 0.92–1.13) or 49–54 hours/week (RR 1.02, 95% CI 0.97–1.07). The certainty of evidence was evaluated as low (very low for 41–48 hours/week).

**Conclusions:**

Daily working hours >12 hours and weekly working hours >55 hours was associated with an increased risk of incidents. The certainty of evidence was low. Hence, further high-quality research is warranted to elucidate these associations.

Extended working hours reduce the opportunity for sleep and restitution. Since sleep is essential for cognitive functioning, restricted sleep or insomnia may diminish alertness and cognitive functions (1–3), thereby increasing the risk of occupational accidents or injuries (4, 5).

Suggested mechanisms for an association between extended working hours and accidents involve sleepiness, fatigue, or disturbance of the circadian rhythm that may weaken the workers’ cognitive performance, vigilance, and decision-making abilities (6, 7).

Knowledge is needed about whether extended working hours affects the risk of accidents, safety incidents, and occupational injuries. The International Labour Organization (ILO) defines excessively long working hours as >48 hours/week and has estimated that 22% of workers worldwide are commonly engaged in such schedules (8). Extended working hours commonly denotes shifts >8 hours/day. In the European Union, 68% of the workers reported working >10 hours/day at least once per month (9).

An occupational injury is defined by the ILO as “any personal injury, disease or death resulting from an occupational accident”. An occupational accident is defined as an “unexpected and unplanned occurrence, including acts of violence, arising out of or in connection with work which results in one or more workers incurring a personal injury, disease or death” (10). Approximately 300 million workplace injuries occur annually (11). In Europe, workplace fatalities represent the third most common cause of death among working men (12). Hence, workplace injuries and fatalities have vast social and economic consequences.

The most recent meta-analysis of extended working hours as a risk factor for occupational injuries and accidents reported that risk rose exponentially beyond the 9^th^ hour on task, and increased substantially for shifts >12 hours (13). Similar conclusions were drawn in earlier reviews (14, 15). These conclusions were based largely on the same original studies, some of which were not published as peer-reviewed articles or lacking statistical analyses. The present systematic review aimed to evaluate critically the certainty of evidence concerning the associations between extended working hours and occupational accidents and injuries. The review was based on studies that met specific quality criteria and we evaluated the certainty of evidence by the Grading of Recommendations Assessment, Development and Evaluation (GRADE) approach (16). This includes an evaluation of the risk of bias, inconsistency, indirectness, imprecision, and of publication bias of the included studies.

The aim of this systematic review was to update the knowledge of the potential consequences of extended working hours on accidents, near-accidents, safety incidents and injuries by considering the overall certainty of evidence.

## Methods

Details of the protocol for this systematic review were registered on PROSPERO and can be accessed at www.crd.york.ac.uk/PROSPERO/display_record.asp?ID=CRD42020134823.

### Literature search

**Figure 1:**
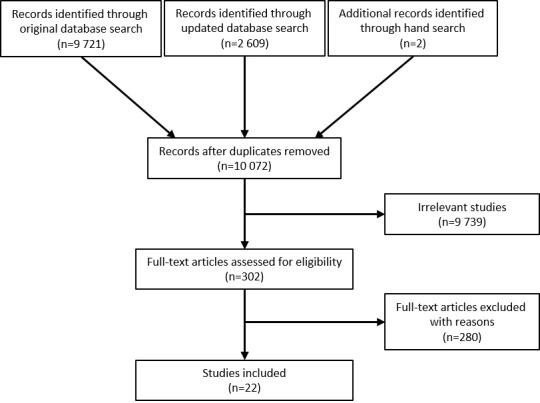
PRISMA flowchart of included studies.

We performed two preliminary searches with search terms, with subsequent investigation of whether relevant key articles on the topic were included. Search terms representing exposure and outcome were chosen after reviewing previously published key articles and reports (13, 17). We searched five databases (Medline, Embase, PsycINFO, Web of Science, and Proquest Health and safety Science Abstract) identifying studies addressing safety incidents associated with working hours, published up to 26 June 2019. An updated search covered studies up to 3 December 2020. In addition, we manually searched the most relevant reviews. The Medline, Embase and PsychINFO search is presented in https://www.crd.york.ac.uk/PROSPEROFILES/134823_STRATEGY_20190625.pdf. Altogether we identified 9721 studies, with the updated search of 2609 studies and manually searched 2 studies, we examined a total of 12 332 studies (figure 1).

### Exposure variables

In the initial search strategy, we included studies with all potential safety-relevant working time patterns as exposure: time of day (shift work, night work), extended working hours (daily or weekly working hours), and shift intensity (number of consecutive shifts and duration of recovery between shifts). We decided to split the search into two systematic reviews. The present review pertains to exposures to extended daily and weekly working hours. A second review will encompass exposures to time of day (shift work, night work, etc.).

### Outcome variables

For an article to be included, the main outcome had to be an explicit measure of an accident, a near accident, a safety incident or an injury attributed to the occurrence of a discrete event. A summary of certainty of evidence was made, based on a sum of incidences of the following four evaluated outcomes: accidents, near-accidents, safety incidents, and injuries. In the following text, these four outcomes are denoted as *incidents*.

### Eligibility criteria

Relevant study designs comprised randomized trials, prospective and retrospective cohort studies, case–control studies and case–crossover studies. Furthermore, the inclusion of papers was limited to peer-reviewed articles containing the following three measurements: (i) an explicit measure of working time exposure; (ii) an explicit measure of the outcome; and (iii), a statistical measure of the association between exposure and outcome. The study context was limited to occupational settings, including adult paid workers aged 18–70 years.

The exclusion criteria were: cross-sectional study design, articles written in non-English language, or studies of non-paid or volunteer workers, military service personnel, or professional athletes.

### Procedure

Initially, two authors independently screened titles and abstracts using Covidence systematic review software (Veritas Health Innovation, Melbourne, Australia, www.covidence.org). At stage two, we evaluated the full texts of potentially eligible records, followed by extraction of data from qualifying studies. We held consensus meetings to resolve disagreements. Figure 1 describes the study selection process. After removal of duplicates, 10 072 articles remained. Of these, 9739 were considered irrelevant based on title and abstract, leaving 302 articles for full text review, of which 280 were excluded, resulting in 22 eligible articles.

### Risk of bias assessment

Two authors independently assessed the risk of bias of each included article in two steps. First, we used a modified checklist scoring system developed at the National Institute of Occupational Health, Norway. For all included papers, the presence of three types of bias was evaluated: *selection bias* (regarding study population, response rate, attrition, etc.), *information bias* (regarding exposure and outcome measurements) and *confounding* (regarding adjustment variables, etc.) (18). Each type of bias was evaluated qualitatively according to a list of specific criteria (see supplementary material www.sjweh.fi/article/3958, table S1) and was categorized as either low, moderate or high. A study obtaining ‘high risk’ on at least one of the three types of bias was rated as having overall high risk of bias, while a study obtaining ‘low risk’ on all three types was considered having overall low risk of bias. The remaining studies were considered as having overall moderate risk of bias.

### Summary measures and synthesis of results

We extracted effect sizes from each study. For studies not reporting effect size as a ratio, we performed calculations into odds ratio (OR) or relative risk (RR) based on information in the article. Results were synthesized in meta-analyses. For the meta-analyses we converted OR and hazard ratios (HR) to RR estimates based on formulas outlined in https://stats.stackexchange.com/questions/130237/convert-hazards-ratio-to-odds-ratio.

### Certainty of evidence

There is no consensus about how to assess certainty of evidence in observational epidemiological studies (19). In this study, we utilized a modified version of the GRADE system (16, 20) to grade the certainty of evidence for an association between extended working hours and incident risk. Evidence was graded for each exposure contrast. We evaluated four exposure contrasts for extended daily working hours: (i) >8 versus ≤8 hours/day, (ii), >12 versus ≤8 hours/day, (iii) overtime versus no overtime, and (iv) >20 hours/day. We likewise defined three exposure contrasts for extended weekly working hours: (i) 41–48 versus 35–40 hours/week, (ii) 49–54 versus 35–40 hours/week, (iii) >55 hours/week versus shorter working weeks. In the GRADE system, certainty of evidence is categorized as either “high”, “moderate”, “low”, or “very low” (16). Since no randomized controlled trials were included in this review, we started at “low” evidence when evaluating the different contrasts. We downgraded the certainty of evidence for the following five reasons: (i) overall risk of bias, (ii) inconsistency, (iii) indirectness, (iv) imprecision, and (v) publication bias. Due to a low number of studies, publication bias was evaluated in a qualitative way, rather than by funnel plots.

### Meta-analyses

We computed random-effects models, which estimate the mean of a distribution of true effects. The random effects model is recommended when there is reason to assume that the true effect vary from one study to the next (21). The Q statistic was computed to assess the heterogeneity of studies (P<0.05 rejects the null hypothesis of homogeneity). The I^2^ statistic shows the heterogeneity in percentages. Meta-analysis was first performed for all studies in a given contrast. Second, meta-analysis was performed after excluding studies with high risk of bias. Our conclusions were based upon the latter. All statistics were carried out using the *meta* function in Stata v.16.1 (StataCorp, College Station, TX, USA).

## Results

### Overview of included studies

Table 1 presents study characteristics of the included 22 studies. The population size ranged from 97–150 438 workers. Study designs included prospective and retrospective longitudinal design, case–control- and case–crossover studies. No randomized trials were identified. The study populations comprised workers in transportation, manufacturing, health care, construction, farming, and the general working population. Exposure and outcome measures were reported at an individual or company level. Some of the studies lack information about the population size or the distribution of age and sex. Data on working hours were either self-reported, registry-based, or reported as a planned change of shift schedule.

**Table 1:** Study characteristics (N=22). [Constr=construction; Gen=general working population; Manuf=manufacturing; Transp=transportation.]

Author, year, country	N	Sector	Study population (sex, composition), working hour arrangement	Study design	Exposure	Exposure assessment	Outcome	Outcome assessment	Risk of bias
Allen et al, 2007 USA (40)	2746	Transp	Truck drivers (mean age 46.3 years, 20.2% women), day work	Prospective study over 4 months	Weekly hours	Registry blended with survey	Acute injury	Survey ^a^	High
Ayas et al, 2006 USA (26)	2737	Health	Medical interns (mean age 28, 53% women), day work after working overnight	Prospective study over 11 months	Daily hours	Survey ^a^	Percutaneous injuries	Survey ^a^	Moderate
Baker et al, 2003, Australia (36)	480	Manuf	Employees in three different sectors in coal mine (no individual data), day and night work	Prospective study over 33 months	Daily hours	Change in shift schedule	Incident frequency	Company records	High
Barger et al, 2005 USA (25)	2737	Health	Medical interns (mean age 28.0 years, 53% women), day and night (24 hours)	Case-crossover and prospective study	Daily hours	Diary	Car crash or near-miss accident after work	Survey partly confirmed by registry	Moderate
Battle & Temblett, 2018 UK (37)	150	Health	Nurses (age and sex not reported, no individual data), day work	Prospective study over 2 years	Daily hours	Change in shift schedule	Injury incident frequency	Company records	High
Härmä et al, 2020 Finland (33)	18 700	Health	Hospital employees (mean age 43 years, 91% women), day, evening and night work	Case-crossover study	Daily and weekly hours	Registry (payroll)	Injury	Registry	Moderate
Landrigan et al, 2004 USA (39)	Health	No information about workers (medical interns), day and night (24 hours)	Prospective study	Weekly hours	Change in shift schedule	Incidence of errors	Registry	High
Larsen et al, 2017 Denmark (29)	150 438	Gen	General working population sample (age 20-59 years, 47% women), adjusted for night work	Retrospective longitudinal study, 1999-2013	Weekly hours	Survey ^a^	Accidental injuries causing hospital contact or death	Registry	Moderate
Lee et al, 2020 Korea (32)	14 484	Gen	General working population sample (age 18-60+ years, 16.5-24.3% women), day work	Retrospective longitudinal study	Weekly hours	Survey ^a^	Occupational fatal accident	Registry	Moderate
Lowery et al, 1998 USA (42)		Constr	Construction workers, aged 15-60+ years, 5% women (no individual data), day work	Retrospective longitudinal study, 1990-94	Daily hours	Registry	Injury incident frequency	Company records	High
Macias et al, 1996 USA (38)		Health	Different groups of workers at an ICU in an American hospital (no individual data), day and night work	Retrospective longitudinal study	Daily hours	Registry	Biological hazards (needlestick, laceration, splash)	Survey ^a^	High
Marcum et al, 2011, USA (27)	1394	Farm	Farmers (age ≥50 years, 49% women), day work	Prospective longitudinal study from 2002-2005	Weekly hours	Survey ^a^	Injury	Survey ^a^	Moderate
Rogers et al, 2004 USA (34)	393	Health	Nurses (mean age 44.8 years, 92% women), day and night work	Prospective study over 4 weeks	Daily hours	Logbook	Errors or near errors	Logbook	High
Scott et al, 2006 USA (35)	502	Health	Critical care nurses (mean age 44.3 years, 92.8% women), day and night work	Prospective study over 4 weeks	Daily hours	Logbook	Errors or near errors	Logbook	High
Soccolich et al, 2013 USA (23)	97	Transp	Truck drivers from three companies (mean age 44 years, 5% women), day and night	Retrospective study over 30 months	Daily hours	Logbook combined with instrumented truck	Safety critical incident rate	Sensor on truck to measure driving	Moderate
Stutts et al, 2003 USA (30)	1403	Transp	Drivers, varying age and sex in cases and controls, day and night	Case-control study	Weekly hours	Survey ^a^	Car crash	Police register	Moderate
Trinkoff et al, 2007 USA (24)	2624	Health	Nurses (age and sex distribution not reported), day and night	Prospective study with follow-up 6 & 15 months later	Daily & weekly hours	Survey ^a^	Needle stick injury	Survey ^a^	Moderate
Weaver et al, 2020 USA (43)	15 276	Health	Medical residents (mean age 28.7–28.9 years, 48-56% women), day and night	Prospective study	Daily & weekly hours	Survey ^a^	Motor vehicle crash and occupational injury	Survey ^a^	High
Vegso et al, 2007 USA (22)	2910	Manuf	Aluminum workers (mean age men 45.9 years, mean age women 43.8 years, no sex distribution), day work	Case-crossover	Daily & weekly hours	Registry	Injury	Company records	Moderate
Wei et al, 2017 USA (41)	2095	Transp	Bus operators (mean age 49 years, 22% women), day and night work	Prospective study from 2006-2011	Daily hours	Registry	Injury	Registry	High
Wong et al, 2014 Canada (28) ^b^	19 131	Gen	General working population (16-55+ years, ~50% women), day and nonstandard (evening, night)	Prospective study, 1999–2004, 2002–2007, 2005–2010	Weekly hours	Survey ^a^	Injury	Survey ^a^	Moderate
Åkerstedt et al, 2002 Sweden (31)^b^	47 860	Gen	National sample, 16-50+ years, ~50% women, day and non-daytime	Repeated national cross-sectional surveys over 20 yrs	Weekly hours	Survey ^a^	Occupational fatal accident	Registry	Moderate

a Includes telephone interview and mailed survey.

^b^ In Wong et al 2014 and Åkerstedt 2002, the lower age range was 16 years. These studies were still included, since the majority of participants in these studies were between 18 and 70.

Most studies assessed the risk of an incident occurring *during* the working hours, however, a few studies assessed risks occurring *after* a work shift (eg, car crashes or patient complications). Most studies (N=5) included both daytime and non-daytime work (evening and/or night work). Seven studies included daytime work only. The overall risk of bias was evaluated as moderate in 12 (22–33) and high in 10 (34–43) studies (supplementary table S2). Exposure measures, outcome measures, effect size metrics, and risk estimates (original and calculated) from each study are shown in supplementary tables S3-A (extended daily working hours) and S3-B (extended weekly working hours). Supplementary tables S4 and S5 show the results of our certainty-of-evidence-evaluation of articles on extended daily and weekly working hours, respectively.

### Extended daily working hours

#### Contrast >8 versus ≤8 hours/day

Six studies investigated associations between >8 versus ≤8 hours/day and risk of safety incidents among workers in healthcare, manufacturing and transportation (supplementary figure S1). One of the six studies were among daytime workers only (22). Based on all six studies, a non-significant elevated risk was observed (RR 1.09, 95% CI 0.91–1.32). No elevated risk was found when analyses were restricted to the three studies showing a moderate risk of bias (RR 0.93, 95% CI 0.72–1.19). One study indicated a beneficial effect of breaks (23).

In conclusion, our analyses did not support an association between working >8 versus ≤8 hours/day, and increased risk of incidents. The conclusion is based primarily on populations working both daytime and non-daytime. The certainty of evidence was low.

#### Contrast ≥12 versus ≤8 hours/day

**Figure 2:**
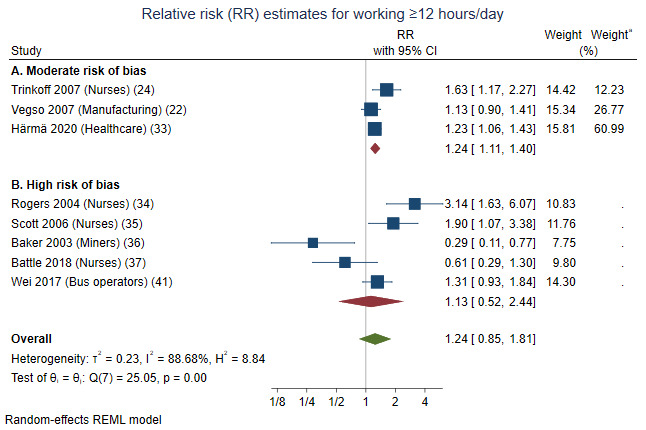
Forest plot of associations between working >12 versus ≤8 hours/day [versus <12 hours/day in Härmä et al (33)] and the relative risk (RR) of incidents, sorted by (A) studies with a moderate risk and (B) studies with a high risk of bias. ^a^ Weights of the studies when moderate-risk-studies are analysed separately.

Eight studies investigated whether working ≥12 versus ≤8 hours/day increased the risk of incidents among workers in healthcare, manufacturing and transportation (figure 2). One of the eight studies were among daytime workers only (22). Based on all eight studies, there was a non-significantly elevated risk (RR 1.24, 95% CI 0.85–1.81). Restricting analyses to the three studies showing a moderate risk of bias, there was a statistically significantly elevated risk (RR 1.24, 95% CI 1.11–1.40).

In conclusion, our analyses supported an association between working >12 versus ≤8 hours/day and increased risk of incidents. The conclusion is based primarily on populations working both day- and non-daytime. The certainty of evidence was low.

#### Contrast overtime versus no overtime

Four studies investigated whether overtime work increases the risk of incidents (supplementary figure S2). Based on all four studies, we observed a non-significantly elevated risk (RR 1.21, 95% CI 0.57–2.57). One of the four studies were among daytime workers only (42). Information on overtime included self-reported working time or data showing overtime pay among transportation-, construction- or healthcare workers. Standard daily working hours were 8 hours (41), 12 hours (34), or not reported (24, 42). A non-significant elevated risk was found in the single study showing a moderate risk of bias (RR 1.08, 95% CI 0.75–1.55) (24). On the other hand, Wei et al (41) reported a beneficial effect of ≥6 hours of overtime work.

In conclusion, our analyses did not support an association between working overtime and increased incidence risk. The conclusion is based primarily on populations working both day- and non-daytime. The certainty of evidence was low.

#### Contrasts >20 versus <12 and ≥24 versus <24 hours/day

Two studies, both having moderate risk of bias, addressed average shift length of >20 and >24 hours respectively, among medical interns. One study reported significantly increased risk for percutaneous injury (RR 1.61, 95% CI 1.46–1.78) when working >20 compared to <12 hours (26). Another study of the same population, reported a significantly increased risk of a vehicle crash (RR 2.30, 95% CI 1.60–3.30) and a near-miss incident (RR 5.81, 95% CI 5.32–6.19) after shifts when working on average ≥24 versus <24 hours (25). Since the two studies were based on the same population, no meta-analysis was performed. In conclusion, our analyses supported an association between working >20–24 hours/day and increased incident risk.

### Extended weekly working hours

#### Contrast 41–48 versus 35–40 hours/week

Eight studies investigated whether working 41–48 versus 35–40 hours/week, increased the risk of incidents among workers in healthcare, farming, manufacturing and among the general working population (supplementary figure S3). Four of the eight studies were among daytime only workers (22, 27, 29, 32). Based on all studies, a non-significantly elevated risk was observed (RR 1.15, 95% CI 0.95–1.39). A non-significantly elevated risk was also found when analyses were restricted to the seven studies showing a moderate risk of bias (RR 1.02, 95% CI 0.92–1.13).

Sensitivity analyses showed similar risk estimates for daytime only workers (RR 1.18, 95% CI 0.90–1.54) and populations working both day- and non-daytime (RR 1.13, 95% CI 0.84–1.53).

In conclusion, our analyses did not support an association between working 41–48 hours/week and increased incidence risk, when compared to 35–40 hours/week, irrespective of whether the populations worked daytime only or both day- and non-daytime. The certainty of evidence was very low.

#### Contrast 49–54 versus 35–40 hours/week

Nine studies investigated whether working 49–54 versus 35–40 hours/week increased the risk of safety incidents among workers in healthcare, transportation, farming, manufacturing, and among the general working population (supplementary figure S4). One of the studies reported risk by cubic regression coefficients and is not included in the figure (40). Four of the nine studies were among daytime only workers (22, 29, 32, 40). Based on the eight studies presenting RR estimates, a non-significantly elevated risk was observed (RR 1.09, 95% CI 0.90–1.32). A non-significantly elevated risk was found when analyses were restricted to the seven studies classified as having a moderate risk of bias (RR 1.02, 95% CI 0.97–1.07). Also the study by Allen et al (40) showed a non-significantly increased risk.

In conclusion, our analyses did not support an association between working 49–54 hours/week and increased incidence risk, when compared to 35–40 hours/week. The conclusion is based primarily on populations working both daytime and non-daytime. The certainty of evidence was low.

#### Contrasts >55 hours/week versus shorter working weeks (variously defined)

Six studies investigated whether working >55 hours/week increased the risk of incidents among workers in healthcare, transportation, and manufacturing (supplementary figure 3). In one of the studies, the risk is reported by cubic regression coefficients, which is not included in the figure (40). Two of the five studies in the meta-analysis included daytime only workers (22, 40). Reference categories were either <40 (22, 30, 33), ≤60 (43) or <63 (39) hours/week. A meta-analysis of the five studies with RR estimates resulted in a significantly elevated risk (RR 1.42, 95% CI 1.06–1.91). The study by Allen et al (40) found a significantly elevated risk for working >60 hours/week. A non-significantly elevated risk was found when analyses were restricted to four studies showing a moderate risk of bias (RR 1.24, 95% CI 0.98–1.57). Weaver (43) tested the dose–response association and found that working 70–80 and >80 (versus ≤70 and ≤80) hours/week were associated with significantly elevated risks (RR 1.41, 95% CI 1.22–1.64, and RR 1.78, 95% CI 1.53–2.07, respectively). Working >80 hours/week was also associated with an increased risk of a motor vehicle crash after work (RR 1.42, 95% CI 1.20–1.68) (43).

**Figure 3:**
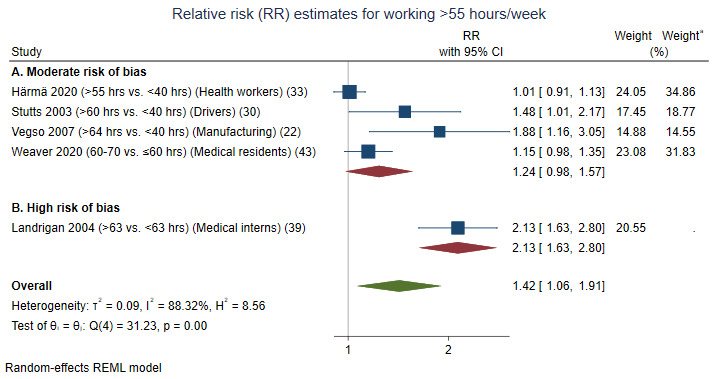
Forest plot of associations between working >55 hours/week and the relative risk (RR) of incidents, sorted by (A) studies with a moderate risk and (B) studies with a high risk of bias. ^a^ Weights of the studies when moderate-risk-studies are analysed separately.

In conclusion, our analyses supported an association between working >55 hours/week and increased incidence risk. The conclusion is based primarily on populations working both day- and non-daytime. The certainty of evidence was low.

### Heterogeneity of studies

For all contrasts, except working >8 hours/day, considerable heterogeneity was observed. Q tests were statistically significant and I^2^ ranged from 85.25% (49–54 hours/week) to 92.80% (41–48 hours/week). For working >8 hours/day, the Q test was not significant and I^2^=24.13%, indicating less heterogeneity. Heterogeneity was acceptable when analyses were restricted to the subgroup of studies with moderate risk of bias. Q tests were non-significant for all contrasts except working >55 hours/week and I^2^ ranged from 0% (49–54 hours/week) to 77.01% (>55 hours/week).

## Discussion

Based on this systematic review and meta-analysis, we found statistically significant associations between working >12 (versus ≤8) and >55 (versus 35–40) hours/week and an elevated risk of incidents. We observed weak or no significant increase of risk associated with >8 work hours/day, or working overtime, when compared to ≤8 work hours/day or no overtime, respectively. Also, we found weak or no significant increase of risk associated with working 41–48 or 49–54 compared to 35–40 hours/week. The majority of the studies included both day- and non-daytime workers. We evaluated the certainty of evidence in all contrasts as “low”, except for the contrast 41–48 versus 35–40 hours/week, which was evaluated as “very low”.

### Comparison to previous reviews

Three previous reviews support the notion that incident risk rise exponentially beyond the 9^th^ hour on task, and increased substantially for shifts >12 hours (13–15). Our analyses corroborate that shifts >12 hours may increase incident risk but do not confirm the assumption of higher risk after the 8^th^ or 9^th^ working hour. A major reason for the low certainty of evidence of many studies was risk of bias (see below). Certainty of evidence was also influenced by imprecision, due to, eg, lack of CI or incomplete information about sampling method or sample size (supplementary tables S4 and S5). Earlier reviews are not readily comparable as they: were mostly based on non-peer-reviewed articles (44, 45), did not include statistical measures of exposure–outcome associations (46), or included cross-sectional studies.

To our knowledge, previous systematic reviews have not addressed the question of effect of overtime on incident risk. Two of the four studies of overtime in the present study show an increase of risk by overtime work. The low certainty of the evidence for this exposure contrast was primarily due to poor definition of overtime, unknown or low sample size, and unknown standard working time. Of the four studies assessing overtime (24, 34, 41, 42), two showed an increasing risk with increasing hours of overtime work (dose–response effect) (supplementary table S3). Similarly, a statistically significant positive increase of risk by increasing working hours was found in three studies of weekly working hours (22, 27, 43). This finding corroborates a risk index model recently published by Fischer et al (13), in which the presented risk map indicates a gradient towards elevated risk with longer working week, particularly during night shifts. Moreover, although working close to or >24 hours is uncommon, two studies on the same population of medical interns indicate that such long shifts increase the risk of both injuries at work and being involved in a car crash (or near-miss incident) after work.

### Risk of bias

A major challenge in this review was to assess the methodological weaknesses, potentially introducing bias. To reveal selection bias, we evaluated whether the participants were representative of the population from which they were drawn with respect to exposure or outcome. Information bias refers to bias arising from misclassification of exposure or outcome, or both. With regard to working time, this may occur if assessed by self-report, due to recall bias or other biases associated with subjective reports. This seems particularly likely if the study hypothesis is not hidden from the participants (lack of blinding). Recall bias may be less problematic when using a logbook (23, 34, 35). Obtaining exposure measurements at the individual level from payroll registries or workforce management systems must be considered the gold standard, enabling accurate and detailed characterization of several dimensions of working time patterns potentially relevant for health (47). Two of the included studies had individual time and date of every shift (22, 33). The third type of potential bias – confounding – is likely to occur if there is inadequate measurement and adjustment of variables that influence both exposure and outcome. Common confounders are age, sex, and socio-economic status. Significant effect modifiers are contents of work (eg, time pressure, multi-tasking), or other types of exposures at work that can affect outcomes. Inadequate adjustment for confounders and effect modifiers, and high risk of selection or information bias may result in either an over- or underestimation of the incident risk and are major contributors to the low certainty of evidence in many of the evaluated contrasts. When restricting meta-analyses to the studies with an overall low or moderate risk of bias, the certainty of evidence may be upgraded. This was the case for all contrasts under study. It is imperative that future studies try to reduce the risk of bias. To expand external validity, further research should also expand to occupational sectors other than the ones included in this review.

### Strengths and limitations of this review

Our review has several strengths. First, we followed all recommended steps of a systematic review, including having a pre-published protocol in which the research question, search protocol, and inclusion criteria were decided *a priori*. Second, we applied a systematic procedure in inclusion and evaluation of each paper and used pre-specified criteria to evaluate the certainty of evidence. Pairs of co-authors independently conducted the procedure and consensus meetings were held to resolve conflicts. Third, we applied GRADE, developed as a transparent approach to grading certainty of evidence. To fit the occupational health field, some documented modifications were made. Finally, cross-sectional studies were excluded due to their inability to infer temporal relationships.

Our systematic review has some limitations. We excluded publications in languages other than English. Second, we did not search grey literature databases or requested unpublished data from principal study authors. Third, although we decided to exclude studies evaluating time of day as exposure (shift work, night work, etc.), the study population in 15 of the 22 studies also includes day- *and* non-daytime workers. Except for one study that adjusted for night work (29), this introduced a circadian component, which may have confounded the effect of long working hours. With the exception of assessing 41–48 versus 35–40 hours/week, we found limited opportunity to take non-daytime work into account as an effect modifier. This complicates interpretation of the findings. As it is not straightforward, in a significant manner, to estimate safety risks associated with extended working hours without taking into account the time-of-day factor, future studies should seek to separate the time of day-component from the extended working hours-component. Fourth, relatively large heterogeneity was observed when considering all included studies in each exposure contrast, indicating substantial differences between the studies. However, restricting analyses to the studies having moderate risk of bias, heterogeneity was non-significant for all contrasts, except one. Upon a qualitative inspection of the forest plots, it is our opinion that the observed heterogeneity is within what is common in this field or research. A fifth limitation concerns the fact that most of the included studies refer to health care workers, ie, the findings may not be representative of other occupations.

### Implications for research and society

With an increasing use of extended working hours in the society, there is a shortage of studies evaluating the effect of such work on health and safety. This systematic review lends some support to an association between extended working hours and increased risk of adverse events among employees and third parties. Since also non-daytime workers were included in 15 of the 22 studies, the conclusions are generalizable primarily for workers in shift or night work. Most of the studies included in this systematic review are, however, hampered by methodological weaknesses, which limit the conclusions that can be drawn. The effect of extended working hours is closely intertwined with other aspects of working time, such as time of day, number of consecutive shifts, breaks and rest-period between two shifts and between two shift periods. Additional information on these aspects, in combination with extended working hours, would increase our insight into the topic and also represent a valuable asset when planning an optimal shift schedule. Although the external validity is somewhat limited due to few studies from the transportation and industrial sectors, it is still likely that the conclusion is valid for several occupations within health care. Future studies should investigate whether associations between working hours and incidents are different between sectors with different work tasks.

### Concluding remarks

The present systematic review and meta-analysis demonstrate associations between working >12 hours/day or >55 hours/week and elevated risk of accidents, near-accidents, safety incidents or injuries, but we consider the certainty of evidence to be low. Hence, further well-controlled prospective studies with objective, detailed exposure data and unbiased outcome assessments are warranted.

## Supplementary material

Supplementary material
