# Supplementary material for: Safety incidents associated with extended working hours. A systematic review and meta-analysis
Source: Scand J Work Environ Health. 2021 Aug 31;47(6):415–24. doi: 10.5271/sjweh.3958 (PMC8504541; doi:10.5271/sjweh.3958)
Supplement: Supplementary material [file SJWEH-47-415-S001.pdf]

Safety incidents associated with extended working hours. A systematic review and meta-analysis[1]

by Dagfinn Matre, PhD,[2] Marit Skogstad, PhD, Tom Sterud, PhD, Karl-Christian Nordby, PhD, Stein Knardahl, PhD, Jan Olav Christensen, PhD, Jenny-Anne S Lie, PhD

1. *Supplementary Material*
2. *Correspondence to: Dagfinn Matre, National Institute of Occupational Health, PB 5330 Majorstuen, 0304* Oslo, Norway. [E-mail: dagfinn.matre@stami.no]

Table S1. Risk of bias assessment criteria

Selection bias criteria
No recruitment selection problems or no non-responder analysis
Specific exclusion criteria
Attrition analysis or discussion of such
High response rate at follow up in longitudinal studies
Information bias criteria
Explicit definition of working hours exposure factors, and representative for period prior to outcome endpoint
Classification of exposure variable on multilevel scale
External observation of exposure variable
Specific case definition
Outcome collected by explicitly described methods of acceptable quality
Outcome based on registry data
Outcome based on examination by third party that is blinded to exposure status
Confounding
Statistical models were appropriate for outcome and measurement of association included confidence intervals
Crude models presented
Number of cases in multivariate analysis was at least 10 times number of independent variables
Study controlled for
Age
Sex
Occupation
Relevant lifestyle factors
Other types of exposure at work
Subjects uninformed by hypotheses tested in study
Each type of bias was assessed qualitatively by evaluating each item in the pre-defined list of criteria presented below. The items had a weighting of two, three or four categories (not shown). Full score on each item within one of the bias categories was evaluated as 'low risk of bias' (category 3 in Table S1), some items with high score and some with low score was evaluated as 'moderate risk of bias' (category 2), whereas low score on all items was evaluated as 'high risk of bias' (category 1). A study obtaining 'high risk' on at least one of the three types of bias was rated as having overall high risk of bias, while a study obtaining 'low risk' on all three types was considered having overall low risk of bias. The remaining studies were considered as having overall moderate risk of bias.

Table S2. Risk of bias assessment including comments

Author, year, country	Selection bias	Information bias	Confounding	Risk of bias	Comment
	3 'low risk', 2 'moderate risk' or 1 'high risk'			High, moderate, low	
Allen et al., 2007 USA (40)	2	1	1	High	Study from a large truck company, combining payroll data on work hours and survey data on injuries. Some riskof selection bias: low response rate, and the final sample constituted 29 % of the eligible sample. The authors report that teh sample was reasonalbly representative. Risk of information bias: limited description and unspecific definition of the outcome variabels (i.e. any short term disability or any acute, musculoskeletal, or other work injury, and unknown number of cases). Multiple testing across several outcomes, and poor presentation further limit the intepretation of the results. No information about time of day incident occured.
Ayas et al., 2006 USA (26)	2	2	2	Moderate	Prospective cohort study of interns in US postgraduate residency programs from July 2002 through May 2003. Case-crossover within-subjects analysis were performed. Misclassification could be a problem since the participants are asked to answer about exposure and outcome at the same time. However, the number of surveys would make this bias and information bias less likely. The number of participants are small compared to the total number of interns, indicating potential selection bias. The proportion of women were larger than that of the total cohort. Workers were aware of study hypothesis.
Baker et al., 2003, Australia (36)	1	2	1	High	Prospective study over 33 months comparing three different working schedules among three different groups of workers in mining; miners, maintenance and coal preparation. The outcome is all sorts of injuries, yielding high risk of information bias. Also some risk of information bias since outcome not confirmed by third party and exposure classified on dichotomous scale. No confounder control. Incident frequency rates of individuals was not available, only at group leve. No information about time of day incident occured. Conclusions can only be generalized from the population under study.
Barger et al,. 2005 USA (25)	2	2	2	Moderate	Prospective nationwide study of residents in hospitals. Survey data on working hours and motor vehicle crash after work verified by a diary on a subsample and insurance claim/police report, respectively. Some risk for selection bias since lack of randomization (i.e. voluntary participation), but age and residency program comparable to register data. Both case-crossover and prospective analyses. Good internal validity, but crude exposure categories and high exposure in the ref. group (24 hours) introduces information bias and limit generalizability of the results.

Battle & Temblett, 2018 UK (37)	1	2	1	High	Prospective study comparing two different work schedules. Small sample size and no information about inclusion criteria. No confouder control e.g. not controlled for age nor lifestyle factors. Self-reported outcome yeidling risk of information bias. No information about time injury occured. Not possible to control whether staff were working overtime, some opted not to work the 12-h shift, during the 12-h shift data collection period, and higher proportion of staff working 12-h shift were full time, indicating potential selection bias. No data on individual workers.
Härmä et al, 2020 Finland (33)	2	3	3	Moderate	A large case-crossover study (> 18 000 hospital employees) to investigate the association of working hours on occupational injuries. Possible problems with selection bias in recruitment. Injury risk was evaluated for the day of the injury, the preeceding day of the injury, and the seven preceding days of the injury day. In a matched-pair interval analysis, a pair of hazard and control intervals contributed by the same participant were compared, based on an assumption of sleep-deficit as a potential pathway. This is as a well-performed study, including the strengths of a large number of participants, the automatic control for between-employee by the cross-over design, and the objective data on exposure and outcome (e.g. registry-data on injuries and daily payroll-data on working hours).
Landrigan et al.,2004 USA (39)	1	2	1	High	Prospective intervention comparing two different work schedules among medical interns. The study was an intervention with randomly assigned participants. Results not generalizable. Possible selection bias since only one hospital studied and no control group. Risk of information bias since underpowered and due to unblind observers, but somewhat reduced since outcome variables were assessed by external individuals. Hour of incident not known. No information about workers available. Weak control for confounders.
Larsen et al, 2017 Denmark (29)	2	3	2	Moderate	Retrospective register-based study over 1.5 years in randomly selected general working population of high quality. Objective data on outcome is a strength. Potential information bias since exposure is assessed by self-report. Potential selection bias since larger companies probably have better-organized systems for reporting accidents. Potential confounding bias since limited information about other occupational demands, education, smoking and weight, but a strenght that occupational code were obtained. Strength that adjusted for sex, age, socioeconomic status and industry and night work.

Lee et al, 2020 Korea (32)	2	3	3	Moderate	A retrospective cohort study of employees in Korea, on the association between long working hours and accidents and suicide mortality. Employers participating in a national nutrition examination survey were matched with the national death registry for the peiod 2007-2016. Cox regression models were developed to estimate hazard ratios, calculating person-days from date of participation in survey until accident, death or end of study. The model adjusted for sex, age, household income, education, occupation and depressive symptoms. Elevated total external cause mortality was found among those who worked 45-52 hrs/week, compared with 35-44 hrs. A higher suicide risk was found for men and women working ≥ 45 hours/week, however no significant associations were found for accident mortality. Limitations include a low number of cases indicating selection bias, a one-time (baseline) self-report of working hours.
Lowery et al, 1998 USA (42)	1	2	2	High	Retrospective cohort study of injuries among construction workers, 1990-1994, based on registry information of injuries from a database of workers' compensation claims. Potential selection bias since no specific inclusion criteria and low follow-up rate. Injuries per 200,000 person-hours estimated. Totally 2,140 injuries included, and estimates shown for lost-work-time (LWT) injuries and non-LWT injurie. Only work-related claims included, and only contracts from companies with known size (high risk selection bias). Risk of information bias is probably low, as information on overtime from registry data. Rate ratios were adjusted for significant variables reducing risk for confounding. No data on individual workers.
Macias et al, 1996 USA (38)	1	2	1	High	Retrospective longitudinal study at an ICU in an American hospital during a 30 months period, studying time of day and duration into shift with respect to exposure to biological hazards in different groups of workers. No individual data. For each hour of the shift, the distribution of 411 different exposures were calculated, as well as the rate of exposure (as a function of number of employees and number of procedures). Results are presented as plots. Some risk of information bias since outcome extracated by survey. A significantly increased risk of hazardous exposures was found for the last 2 hours of a 12 hour shift. Confidence intervals plotted, however not stated. Further limitations: High risk of selection bias since only one hospital was included, no adjustment for potential confounders (age, gender, occupation). No data on individual workers.
Marcum et al, 2011, USA (27)	2	2	2	Moderate	Prospective longitudinal study of farmwork-related injury, four times questionnaire by telephone or mail. A number of risk factors investigated. Injuries were included if they were farmwork-related, non-fatal, resulting in need to see a doctor, or if unable to work for ≥ half a day. Possible problems with selection bias in recruitment. Potential information bias since injuries were based on self-report with no specific guidelines. In the multivariable analysis, a 10 years increase of age corresponded to 19% decreased OR of farmwork-related injury. Less than 20% of participants were working 40+ hours/week. Counfounding possible since information about weight was not measured at each wave. No information about time of day incident occured.

Rogers et al., 2004 USA (34)	2	1	2	High	Randomized sample, but a rather low response rate induced risk for selection bias. Socio-demografic variables reported to be rather representative. Combining survey data and logbooks on hours worked and occurence of errors. Some risk of information bias since, working hours and accidents based on self-reprting during a 4-week period. Statistical analyses not adequate. Confidence intervals not reported. Adjustment not described in table, hence confounding bias cannot be ruled out. No information about time of day incident occured.
Scott et al., 2006 USA (35)	2	1	2	High	Randomized sample, but a rather low response rate induced risk for selection bias. Socio-demografic variables reported to be rather representative. Combining survey data and logbooks on hours worked and occurence of errors. Some risk of information bias since, working hours and accidents based on self-reprting during a 4-week period. Statistical analyses not adequate. Confidence intervals not reported. Adjustment not described in table, hence confounding bias cannot be ruled out. No information about time of day incident occured.
Soccolich et al., 2013 USA (23)	2	2	2	Moderate	Naturalistic study of truck drivers, safety critical incidents evaluated by sensors on the truck, acktivity during shift was self-reported. Convenience sample of companies and drivers. A main effect of work hours as a continous measure on injury rate was obsereved. Pairwise comparison of injury risk for each hour worked (1-11) is a strength. Multiple testing with regard to different exposure classification was performed, but limited information about statistical uncertainty and testing. Potential multiple comparisons problem. No adjusment for confounding (mainly men 97%, but variation in age and experience).
Stutts et al., 2003 USA (30)	2	2	2	Moderate	Case-control study of police registered car crashes from a single state. About 55 % of car crashes and 61% of controls were successfully interviewed a month after incident. Self-reported data on exposure after incident. Sleep relatedness based on police officers’ evaluation may, however, be biased if information about long working hours was used as criterion for categorization of accident as sleep-related. Analyses adjusted for driver’s age and gender. The study has two control groups; non-crash controls and a control group of non-sleep related crashes. The latter comparison group was considered most valid, and the estimates is based on this comparison. The choice of reference category 40-49 hours, may include workers with rather long work days. No information about time of day when incident occurred.
Trinkoff et al., 2007 USA (24)	3	2	2	Moderate	A three-wave longitudinal suvey among randomly selected nurses in two states, USA, with a high participation rate at wave 2 & 3. Data was collected on job schedule, job demands, daily frequency of needle use. Self-report of needle stick injury, both before and during follow-up. Risk of needle stick injury in the past year was reported as age-adjusted OR, and only nurses with one year work in the present position at inclusion was included in the study. Risk of information bias since self-reported survey data. Weak confounder control. No information about time of day incident occured.

Weaver et al, 2020 USA (43)	1	3	2	High	A nationwide prospective cohort study among first-year resident physicians, to investigate the effect of implementation of a 16-h limit on consecutive hours of work in 2011 (ACGME), including 5 years prior to 2011 and 3 years after. Monthly reports by participants of hrs of work, extended duration shifts and adverse safety outcomes ; including motor vehicle crashes, percutaneous injuries and attentional failures. Analyses comparing the incidence of each outcome before and after 2011 were performed using generalized linear models, adjusted for potential confounders. Limitations: Only 13% of the invited subjects participated, unspecified exclusion criteria and no non-responder or attrition analyses are mentioned, thus selection bias is probable. Strengths include the large size of the cohorts; the adjustment for relevant variables (age, gender, BMI, and specialty), and the finding of an association between extended duration shifts and prolonged weekly work hours and an increased risk of adverse safety outcomes in both cohorts. No crude models or adjustment for other exposures at work indicate potential risk for confounding.
Vegso et al., 2007 USA (22)	3	2	2	Moderate	Case-crossover study among manufacturing workers related to hours worked during pervious week, contrasted with hours worked prior to a non-injury shift. Work hours per day was obtained from payroll data, which is a strength. Weak statistical test to assess difference between weeks, but logistic regression determined dose-response. Potential under-reporting of injuries to company is a potential source of bias.
Wei et al., 2017 USA (41)	3	1	2	High	Prospective study of bus drivers' injury risk using data from the bus company. Cox regression with repeated events was used to analyse associations between the shift pattern that a driver was allocated to and the risk of injury in the work as a bus driver. Lowered risk for confounding bias since adjustments were made for no of working years, operator characteristics, and work exposures: age, gender, work years, job classification, number route of driving and route type. A driver could change the actual work pattern during the period of shift assignment, thus opening up for misclassification of exposure. The exposure contrast is very weak, since only 1.5% of events occured within the highest exposure stratum of >=12 hours daily work. No information about time of day of accident.
Wong et al, 2014 Canada (28)	2	2	2	Moderate	Prospective study of work injuries over 6 years in general working population switching between work schedules. Strengths are high response rate and fully adjusted models (region, occupational charactristics, no employees). Potential selection bias since required participation in all 6 waves may give selection of highly motivated participants. Potential information bias since asked about exposure and outcome at same time. Potential confounding bias since limited information about occupational demands, smoking and weight. No information about time of day of accident.

Åkerstedt et al, 2002 Sweden (31)	2	2	2	Moderate	Repeated national cross-sectional surveys of fatal accidents. Low risk of information bias concerning outcome since fatality from national registry is outcome. Low risk of selection bias since high response rate, and representative sample. Potential risk of information bias regarding exposure since crude estimate of working hours, and unspecific period. Potential confounding since not controlled for medication, lifestyle factors and for diagnoses causing sleep disturbances. No information about time of day of accident.

Table S3. Effects of A) extended daily working hours and B) extended weekly working hours on safety incidents, data from individual studies

Study	Working hour exposure	Working hour arrangement	Outcome	Statistical metric	OR, RR, HR	95% CI low	95% CI high	Comment	Calculated RR[d]			
**A. Extended daily working hours**									r or P0[b]	RR	95% CI low	95% CI high
Ayas et al., 2006 (26)	<12 hours	20-hour or more schedule	Injury	OR	1 Ref.							
	≥20 hours				**1.61**	**1.46**	**1.78**		0.001	1.61	1.46	1.78
Baker et al., 2003 (36)	8 hours (7 days)	Rotating 8-hour/7-day roster (day, night, afternoon)	Incident	RR[a]	1 Ref.							
	12 hours (7 days), mining	12-hour/7-day roster (day, night)			1.22	0.73	2.10					
	12 hours (7 days), maintenance				1.27	0.76	1.12					
	12 hours (7 days), coal preparation				**0.28**	**0.11**	**0.76**					
Barger et al,. 2005 (25)	<24 hours	24-hour schedule		OR	1 Ref.							
	≥24 hours		Car crash after work		**2.30**	**1.60**	**3.30**		0.0004	2.30	1.60	3.30
	≥24 hours		Near-miss incident		**5.90**	**5.40**	**6.30**		0.006	5.81	5.32	6.19
Battle & Temblett, 2018 (37)	8 hours	8-hour or 12-hour day shift schedule	Injury	RR[a]	1 Ref.							
	12 hours				0.61	0.29	1.30					
Härmä et al, 2020 (33)	<12 hours	Rotating 3-shift schedule (day, evening, night)	Injury	OR	1 Ref.							
	≥12 hours				**1.23**	**1.06**	**1.42**		0.00001	1.23	1.05	1.41
Lowery et al, 1998 (42)	No overtime	Daytime schedule	Injury	RR	1 Ref.							
	>0-20% overtime				1.20	0.88	1.64					
	>20% overtime				**1.57**	**1.13**	**2.17**					

Macias et al, 1996 (38)	8 hours	Shifts ≤12-hours during day or night	Injury	RR[a]	1 Ref.						
	9 hours				1.02	0.78	1.30				
	10 hours				1.16	0.92	1.45				
	11 hours				1.02	0.79	1.30				
Rogers et al., 2004 (34)	≤8.5 hours	Non-defined schedule, 24-hour operations	Errors	OR[a]	1 Ref.						
	>8.5 hours				1.85	0.97	3.52	0.016	1.83	0.97	3.38
	≥12.5 hours				**3.29**	**1.62**	**6.67**	0.020	3.14	1.60	5.98
	≤8.5 hours + overtime				1.34	0.67	2.70				
	>8.5 hours + overtime				1.53	0.62	3.76				
	≥12.5 hours + overtime				**3.26**	**1.43**	**7.43**	0.017	3.14	1.42	6.71
Scott et al., 2006 (35)	≤8.5 hours	Non-defined schedule, 24-hour operations	Errors	OR[a]	1 Ref.						
	>8.5 hours				1.42	0.74	2.74	0.020	1.41	0.74	2.65
	≥12.5 hours				**1.94**	**1.07**	**3.53**	0.020	1.90	1.06	3.36
Soccolich et al., 2013 (23)	≤8	Primarily day time schedule, may extend into night	Safety-critical events	OR	1 Ref.						
	>10				0.70	0.43	1.13	0.088	0.72	0.45	1.12
Trinkoff et al., 2007 (24)	<8 hours	Day and non-daytime schedule	Injury	OR	1 Ref.						
	9-11 hours				1.19	0.88	1.61	[c]	0.92	0.64	1.32
	>12 hours				**1.68**	**1.27**	**2.22**	[c]	1.63	1.17	2.26
	Continuous per hour				**1.10**	**1.05**	**1.16**				
	≥13 hours (least once a week)				**1.66**	**1.28**	**2.15**				
	Mandatory overtime				1.25	0.93	1.85				
Vegso et al., 2007 (22)	<8 hours	Daytime schedule	Injury	HR	1 Ref.						
	8-12 hours				1.18	0.79	1.84	0.00001	1.18	0.79	1.84
	>12 hours				1.13	0.91	1.42	0.00001	1.13	0.91	1.42
Wei et al., 2017 (41)	<7 hours	Non-defined schedule, 24-hour operations including split shifts	Injury	HR	**4.60**	**3.80**	**5.50**				

	7-12 hours				1 Ref.							
	≥12 hours				1.40	0.90	2.20		0.3	1.31	0.92	1.81
	<3 hours overtime				1.10	0.80	1.40					
	3 - <6 hours overtime				0.90	0.70	1.30					
	≥6 hours overtime				0.40	0.20	0.70		0.3	0.44	0.23	0.74

B. Extended weekly working hours

Allen et al., 2007 (40)	<40 hours	Daytime schedule	Injury	b	-0.016	-0.041	0.009	p=0.204				
	48.10-60 hours				0.003	-0.024	0.030	p=0.852				
	>60 hours				**0.074**	**0.027**	**0.121**	**p=0.0024**				
Härmä et al, 2020 (33)	≤40 hours	Rotating 3-shift schedule (day, evening, night)	Injury	OR	1 Ref.							
	>40 hours				0.99	0.94	1.04		0.00001	0.99	0.94	1.04
	>48 hours				1.00	0.92	1.08		0.00001	1.00	0.92	1.08
	>55 hours				1.01	0.91	1.13		0.00001	1.01	0.91	1.13
Landrigan et al., 2004 (39)	>63 hours vs. ≤63 hours	24-hour schedule	Medical errors	OR[a]	**2.13**	**1.63**	**2.80**		0.14	1.85	1.50	2.25
			Preventable adverse events		1.82	0.96	3.45					
			Intercepted serious errors		**1.90**	**1.33**	**2.72**					
			Non-intecepted serious errors		**2.31**	**1.44**	**3.69**					
Larsen et al, 2017 (29)	32-40 hours	Risk adjusted for night work	Accidental injuries causing hospital contact or death	RR	1 Ref.							

	41-48 hours				0.96	0.9	1.01					
	>48 hours				1.02	0.95	1.09					
Lee et al, 2020 (32)	35-44 hours	Daytime schedule		HR	1 Ref.							
	45-52 hours				1.78	0.57	5.52		0.0003	1.78	0.57	5.52
	>52 hours				0.98	0.32	2.98		0.0003	0.98	0.32	2.98
Marcum et al, 2011 (27)	<40 hours	Daytime schedule	Injury	OR	1 Ref.							
	>40 hours				**1.27**	**1.21**	**1.34**		0.109	1.72	0.86	3.12
Rogers et al., 2004 (34)	<40 hours	Non-defined schedule, 24-hour operations	Errors	OR[a]	1 Ref.							
	>40 hours				**1.96**	**1.40**	**2.74**		0.086	1.81	1.35	2.39
	<50 hours				1 Ref.							
	>50 hours				**1.92**	**1.39**	**2.66**		0.101	1.78	1.34	2.33
Stutts et al., 2003 (30)	<40 hours	Day, evening, night schedule	Car crash outside work	OR	0.77	0.5	1.19					
	40-49 hours				1 Ref.							
	50-59 hours				1.17	0.77	1.78		0.094	1.15	0.79	1.66
	>60 hours				**1.48**	**1.01**	**2.18**		0.094	1.42	1.01	1.96
Trinkoff et al., 2007 (24)	≤40 hours	Day and non-daytime schedule	Injury	OR	1 Ref.							
	41-49 hours				0.85	0.62	1.17		[c]	1.1	0.77	1.56
	≥50 hours				1.12	0.80	1.57		[c]	0.7	0.44	1.12
	Continuous per hour				1.01	1.00	1.02	**Trend: p=0.04**				
Vegso et al., 2007 (22)	<40 hours	Daytime schedule	Injury	HR	1 Ref.							
	40-48 hours				**1.26**	**1.03**	**1.54**		0.00001	1.26	1.03	1.54
	49-56 hours				1.19	0.92	1.52		0.00001	1.19	0.92	1.52
	57-64 hours				1.21	0.84	1.75					
	>64 hours				**1.88**	**1.16**	**3.05**		0.00001	1.88	1.16	3.05
	Continuous per hour											
Weaver et al., 2020 (43)	≤60 hours	24-hour schedule	Motor vehicle crash	RR	1 Ref.							

	>60 & ≤70 hours				1.14	0.97	1.33					
	>70 & ≤80 hours				1.17	1.00	1.37					
	>80 hours				**1.42**	**1.20**	**1.68**	**Trend: p<0.001**				
	≤60 hours		Percutaneous injury	RR	1 Ref.							
	>60 & ≤70 hours				1.15	0.98	1.36					
	>70 & ≤80 hours				**1.41**	**1.22**	**1.64**					
	>80 hours				**1.78**	**1.53**	**2.07**					
Wong et al, 2014 (28)	1-25 hours	Day and nonstandard (evening, night) schedule	Injury	HR	**1.35**	**1.08**	**1.69**					
	26-35 hours				**1.30**	**1.11**	**1.53**					
	36-40 hours				1 Ref.							
	>41 hours				0.88	0.74	1.04		0.118	0.89	0.75	1.04
Åkerstedt et al, 2002 (31)	<50 hours	Day and non-daytime schedule	Death	RR	1 Ref.							
	>50 hours				0.78	0.34	1.82					

[a]Risk estimate and confidence interval calculated based on crude data in original study.

[b]Calculated RR

From Hazard ratio (HR):

Formula: $RR=(1-e^{HR\ln(1-r)})/r$

where HR is the hazard ratio and r is the rate for the reference group. See Shor et al. (2017) doi: 10.1016/j.socscimed.2017.05.049

From odds ratio (OR):

$RR=OR/(1-Po)+(Po\cdot OR)$

where Po is the incidence of the outcome of interest in the non-exposed group.

[c] RR values given in article, in addition to OR

Values in bold are statistically significant at p<0.05. Ref: Reference

Table S4, assessing the certainty of evidence between extended daily working hours and incident risk

The tables below were adapted from "How to GRADE the quality of the evidence", appendix 1

(https://cgf.cochrane.org/sites/cgf.cochrane.org/files/public/uploads/uploads/how_to_grade.pdf)

One table was filled out for each of four exposure contrasts for extended daily working hours: (i) >8 hours/day vs. ≤8 hours/day, (ii), >12 hours/day vs. ≤8 hours/day, (iii) overtime vs. no overtime, and (iv) >20 hours/day.

GRADE criteria	Rating	All studies, 6	Low or moderate risk studies, 3
**Exposure: >8 hours/day**			
**Study design**	RCT (starts as high quality)<br>**Non-RCT (starts as low quality)**		
**Risk of bias**	No<br>Serious (-1)<br>Very serious (-2)	-1	0
**Inconsistency**	No<br>Serious (-1)<br>Very serious (-2)	0 overlapping CI, point estimates similar	0 overlapping CI, point estimates similar
**Indirectness**	No<br>Serious (-1)<br>Very serious (-2)	-1 for differences in populations	-1 for differences in populations
**Imprecision**	No<br>Serious (-1)<br>Very serious (-2)	-1 few participants, wide CI	0
**Publication bias**	Undetected<br>Strongly suspected (-1)	0	0
**Other**	Large effect (+1 or +2)<br>Dose response (+1 or +2)<br>No plausible confounding (+1 or +2)	+1 dose-response in 3 studies	+1 dose-response in 3 studies
**Certainty of evidence**		Very low	Low

GRADE criteria	Rating	All studies, 9	Low or moderate risk studies, 3
**Exposure: >12 hours/day**			
**Study design**	RCT (starts as high quality)<br>**Non-RCT (starts as low quality)**		
**Risk of bias**	No<br>Serious (-1)<br>Very serious (-2)	-1	0
**Inconsistency**	No<br>Serious (-1)<br>Very serious (-2)	-1, two of 7 studies show reduced risik, remaining increased risik	0 overlapping CI, point estimates similar
**Indirectness**	No<br>Serious (-1)<br>Very serious (-2)	-1 for differences in populations	-1 for differences in populations
**Imprecision**	No<br>Serious (-1)<br>Very serious (-2)	-1 few participants	0
**Publication bias**	Undetected<br>Strongly suspected (-1)	0	0
**Other**	Large effect (+1 or +2)<br>Dose response (+1 or +2)<br>No plausible confounding (+1 or +2)	+1 dose-response in 3 studies	+1 dose-response in 3 studies
**Certainty of evidence**		Very low	Low

GRADE criteria	Rating	All studies, 4	Low or moderate risk studies, 1
**Exposure: overtime**			
**Study design**	RCT (starts as high quality)<br>**Non-RCT (starts as low quality)**		
**Risk of bias**	No	-1	0

	Serious (-1)<br>Very serious (-2)		
**Inconsistency**	No<br>Serious (-1)<br>Very serious (-2)	0	0
**Indirectness**	No<br>Serious (-1)<br>Very serious (-2)	-1 for differences in populations	0
**Imprecision**	No<br>Serious (-1)<br>Very serious (-2)	-1 few participants	0
**Publication bias**	Undetected<br>Strongly suspected (-1)	0	0
**Other**	Large effect (+1 or +2)<br>Dose response (+1 or +2)<br>No plausible confounding (+1 or +2)	+1 dose-response in 3 studies	0
**Certainty of evidence**		Very low	Low

GRADE criteria	Rating	All studies, 2	Low or moderate risk studies, 2
**Exposure: >20 hours/day**			
**Study design**	RCT (starts as high quality)<br>**Non-RCT (starts as low quality)**		
**Risk of bias**	No<br>Serious (-1)<br>Very serious (-2)		0
**Inconsistency**	No<br>Serious (-1)<br>Very serious (-2)		-1, for reference contrasts difficult to compare
**Indirectness**	No<br>Serious (-1)<br>Very serious (-2)		
**Imprecision**	No		0

	Serious (-1)<br>Very serious (-2)		
**Publication bias**	Undetected<br>Strongly suspected (-1)		0
**Other**	Large effect (+1 or +2)<br>Dose response (+1 or +2)<br>No plausible confounding (+1 or +2)		+1 effect size >2
**Certainty of evidence**			Low

Table S5, assessing the certainty of evidence between extended weekly working hours and incident risk

The tables below were adapted from "How to GRADE the quality of the evidence", appendix 1

(https://cgf.cochrane.org/sites/cgf.cochrane.org/files/public/uploads/uploads/how_to_grade.pdf)

One table was filled out for each of three exposure contrasts for extended weekly working hours: (i) 41-48 hours/week vs. 35-40 hours/week, (ii) 49-54 hours/week vs. 35-40 hours/week, (iii) >55 hours/week.

GRADE criteria	Rating	All studies, 8	Low or moderate risk studies, 6
**Exposure: 41-48 hours/week**			
**Study design**	RCT (starts as high quality)<br>**Non-RCT (starts as low quality)**		
**Risk of bias**	No<br>Serious (-1)<br>Very serious (-2)	0	0
**Inconsistency**	No<br>Serious (-1)<br>Very serious (-2)	0	0
**Indirectness**	No<br>Serious (-1)<br>Very serious (-2)	-1, different populations	-1, different populations
**Imprecision**	No<br>Serious (-1)<br>Very serious (-2)	-1, low sample size non-overlapping CI	-1
**Publication bias**	Undetected<br>Strongly suspected (-1)		
**Other**	Large effect (+1 or +2)<br>Dose response (+1 or +2)<br>No plausible confounding (+1 or +2)	+1, pos trend	+1 pos trend
**Certainty of evidence**		Very low	Very low

GRADE criteria	Rating	All studies, 9	Low or moderate risk studies, 7
	**Exposure: 49-54 hours/week**		
**Study design**	RCT (starts as high quality)<br>**Non-RCT (starts as low quality)**		
**Risk of bias**	No<br>Serious (-1)<br>Very serious (-2)	0	0
**Inconsistency**	No<br>Serious (-1)<br>Very serious (-2)	0	0
**Indirectness**	No<br>Serious (-1)<br>Very serious (-2)	-1, different populations	-1, different populations
**Imprecision**	No<br>Serious (-1)<br>Very serious (-2)	0	0
**Publication bias**	Undetected<br>Strongly suspected (-1)		
**Other**	Large effect (+1 or +2)<br>Dose response (+1 or +2)<br>No plausible confounding (+1 or +2)	+1, pos trend, stor sample	+1, pos trend, stor sample
**Certainty of evidence**		Low	Low

GRADE criteria	Rating	All studies, 6	Low or moderate risk studies, 8
	**Exposure: >55 hours/week**		
**Study design**	RCT (starts as high quality)<br>**Non-RCT (starts as low quality)**		
**Risk of bias**	No<br>Serious (-1)<br>Very serious (-2)	-1	0

**Inconsistency**	No<br>Serious (-1)<br>Very serious (-2)	-1	0
**Indirectness**	No<br>Serious (-1)<br>Very serious (-2)	-1, different populations	-1, different populations
**Imprecision**	No<br>Serious (-1)<br>Very serious (-2)	0	0
**Publication bias**	Undetected<br>Strongly suspected (-1)		
**Other**	Large effect (+1 or +2)<br>Dose response (+1 or +2)<br>No plausible confounding (+1 or +2)	+1, pos trend, stor sample	+1, pos trend, stor sample
**Certainty of evidence**		Very low	Low

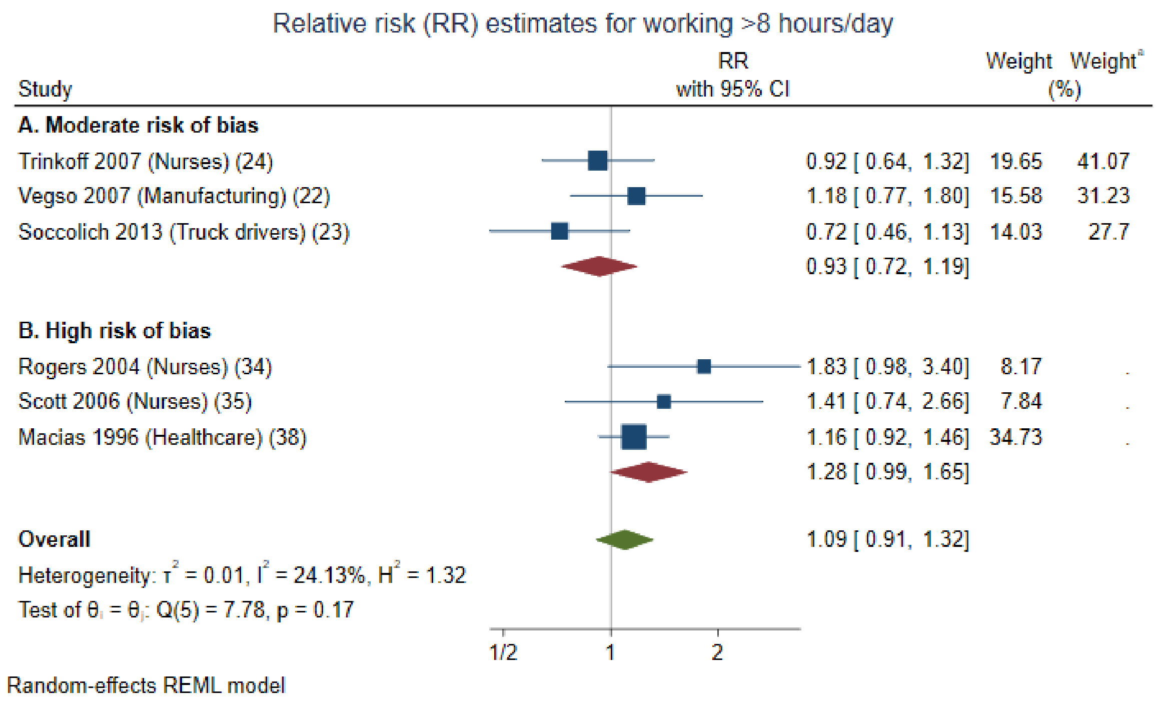

Figure S1. Forest plot of associations between working more than 8 hours/day vs. ≤8 hours/day, and relative risk (RR) of incidents, sorted by A) studies with a moderate, and B) studies with a high risk of bias. [a]weights of the studies when moderate-risk-studies are analysed separately.

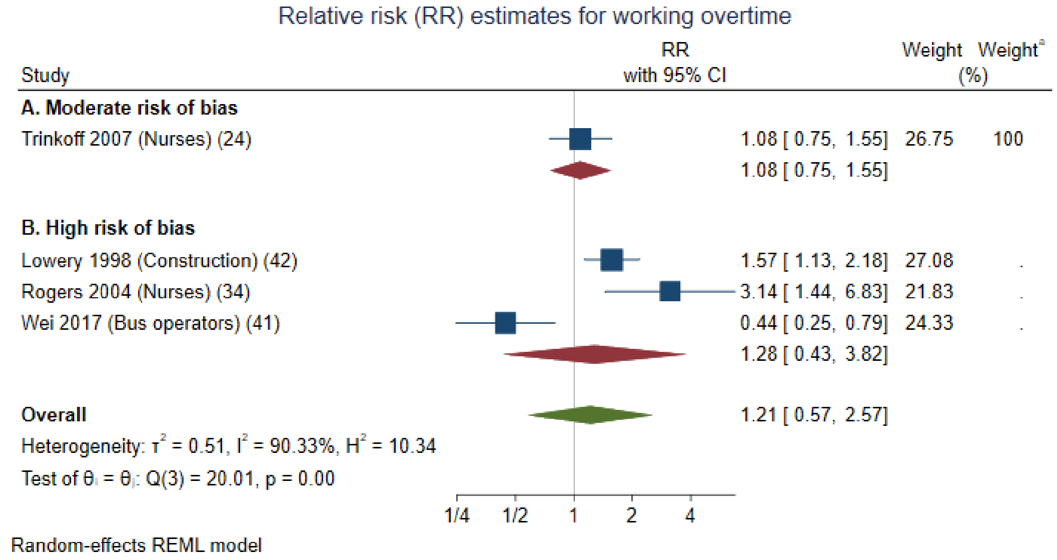

Figure S2. Forest plot of associations between working overtime and the relative risk (RR) of incidents, sorted by A) a study with a moderate-, and B) studies with a high risk of bias. [a]weights of the studies when moderate-risk-studies are analysed separately.

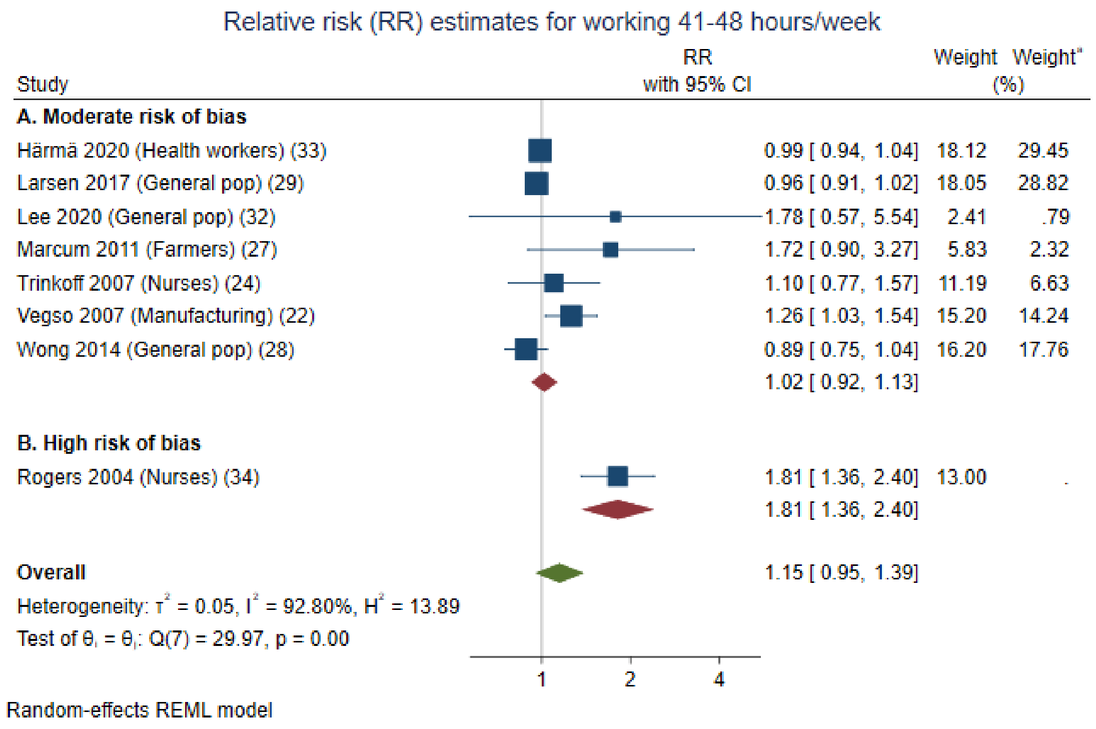

Figure S3. Forest plot of associations between working 41-48 hours/week, vs. 35-40 hours/week, and the relative risk (RR) of incidents, sorted by A) studies with a moderate- and B) studies with a high risk of bias. [a]weights of the studies when moderate-risk-studies are analysed separately.

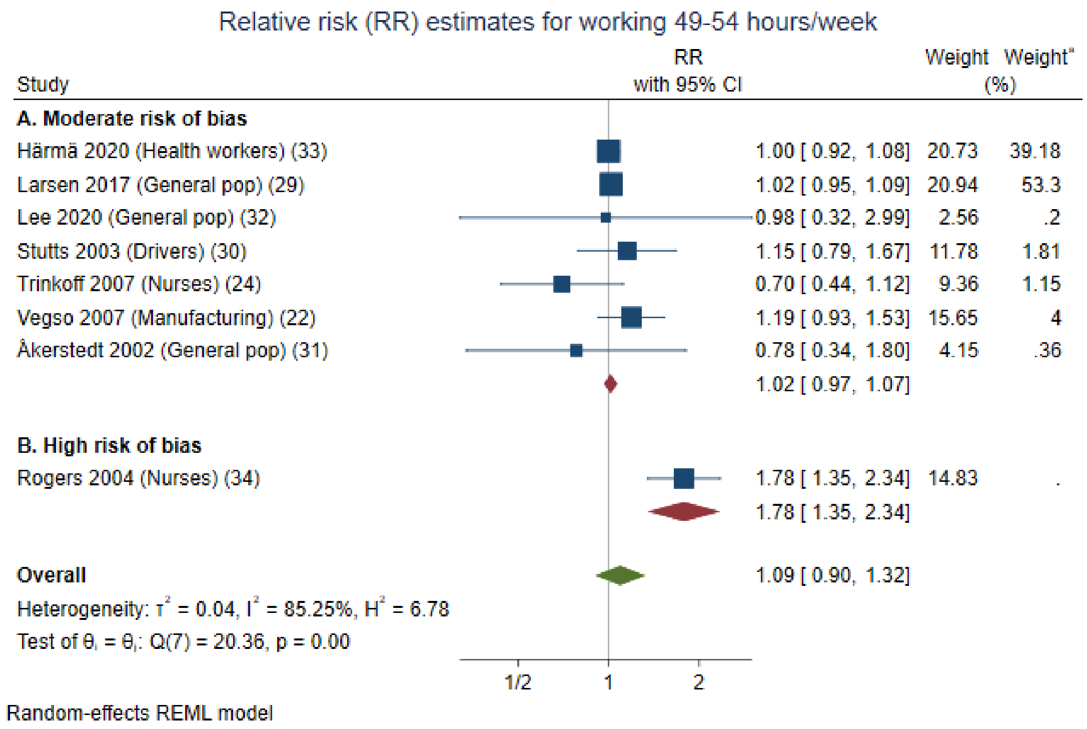

Figure S4. Forest plot of associations between working 49-54 hours/week, vs. 35-40 hours/week, and the relative risk (RR) of incidents, sorted by A) studies with a moderate- and B) studies with a high risk of bias. [a]weights of the studies when moderate-risk-studies are analysed separately.
